# The effects of treadmill exercise on brain angiogenesis in ovariectomized rats

**DOI:** 10.14814/phy2.15864

**Published:** 2023-11-14

**Authors:** Eun‐Jung Yoon, Jiwon Jeong, Eunji Yoon, Dongsun Park

**Affiliations:** ^1^ Laboratory of Animal Physiology and Medicine, Department of Biology Education Korea National University of Education Cheongju Korea

**Keywords:** angiogenesis, brain, exercise, menopause, ovariectomy

## Abstract

Menopause is associated with vascular dysfunction attributed to reduced estrogen levels. Exercise has been proposed to promote angiogenesis and vascular dysfunction. However, studies of brain angiogenesis during menopause are limited. We analyzed the effects of exercise on angiogenesis‐related factors in menopausal rat model. Twenty‐week‐old female Sprague–Dawley rats (*N* = 18) were randomly divided into a normal control group (N, *n* = 6), an ovariectomized control group (OVX, *n* = 6), and an ovariectomy + exercise group (OVX‐EX, *n* = 6). Treadmill exercises were conducted in the OVX‐EX group for 8 weeks (15–60 m/min, 1 h/day, and 5 days/week). The current study showed that the expression of angiogenesis‐related factors (platelet‐derived growth factor subunit A, B, vascular endothelial growth factor, angiopoietin 1, and angiopoietin 2) significantly decreased in the cortex of the OVX group. However, these factors were significantly restored in the cortex of the OVX‐EX group after 8 weeks of treadmill exercise. In summary, estrogen deficiency causes vascular dysfunction by inhibiting the expression of angiogenesis‐related factors. However, exercise can restore angiogenesis‐related factors in OVX rats. Exercise eventually prevents vascular dysfunction in the brain and may help prevent cognitive dysfunction in menopausal women.

## 1 INTRODUCTION

Menopause is a natural phenomenon caused by aging, leading to a decline in estrogen levels as the ovary's function deteriorates (Horstman et al., 2012). Menopausal women experience drastic physical changes, as reduced estrogen secretion diminishes the expression of angiogenic factors (Trenti et al., 2018). Consequently, estrogen deficiency results in a series of alterations, such as dyslipidemia, vascular endothelial cell dysfunction, and increased vascular stiffness (Gavin et al., 2009; Nair et al., 2021). Various methods, including medication and hormone replacement therapy, have been proposed to alleviate these symptoms, but they may carry several side effects. Exercise, on the other hand, is considered to have fewer adverse effects and has been shown to increase the expression of angiogenic factors, influencing angiogenesis (Kwak et al., 2018). These angiogenesis factors encompass platelet‐derived growth factor (PDGF), vascular endothermic growth factor (VEGF), and angiopoietin (Ang). Exercise induces hypoxic conditions, triggering compensatory mechanisms that lead to increased expression of these factors and, subsequently, angiogenesis (Messmer‐Blust et al., 2009; Suhr et al., 2021).

PDGF stimulates vascular endothelial cells to induce angiogenesis and exists as dimers (AA, AB, and BB) connected by two polypeptide chains, A and B, via disulfide bonds (Abboud, 1995; Cho MI, 1995). Estrogen levels also influence PDGF, with insufficient estrogen leading to reduced PDGF expression. However, exercise enhances PDGF expression, as demonstrated in both animal and human studies, particularly in skeletal muscle (Czarkowska‐Paczek et al., 2006, 2011).

During exercise, VEGF levels increase (Maass et al., 2016). VEGF plays a crucial role in regulating angiogenesis, promoting the growth of new blood vessels from existing ones vascular endothelial cells production, and positively impacting cognitive function by participating in the generation, regeneration, and recovery of new neural cells (Melincovici et al., 2018). Hypoxia rapidly and reversibly increases VEGF expression through the transcription and stability of VEGF mRNA, primarily mediated by hypoxia‐inducible factor‐1 (Forsythe et al., 1996). The increase in VEGF during hypoxia can be considered as a natural compensatory response to the augmented blood flow through the proliferation of blood vessels, which is also expected during exercise (Li et al., 2020; Steinbrech et al., 1999).

Angiogenesis is also induced by the expression of Angs, Ang1 involved in blood vessel stability, and Ang2 increasing blood vessel permeability. Histological tests have shown that Ang1 plays a pivotal role in creating and maintaining mature blood vessels by recruiting peripheral cells from vascular endothelial cells in the body (Arita et al., 2014). Ang2 mRNA is predominantly expressed in vascular endothelial cells and expression is upregulated in hypoxic conditions, such as exercise, as well as in response to VEGF, basic fibroblast growth factor, tumor necrosis factor alpha, angiotensin II, and leptin (Davis et al., 1996; Gale et al., 2002; Koh et al., 2002; Maisonpierre et al., 1997). In a previous study, the expression of Ang1 and Ang2 in skeletal muscles was significantly increased after performing resistance exercises in healthy young men (Gavin et al., 2007).

Despite numerous studies indicating the influence of exercise on angiogenesis, most of the research has been focused on skeletal muscle, and limited investigations have been conducted on estrogen or menopausal models. Therefore, this study aims to investigate the expression of angiogenesis‐related factors in the brains of ovariectomized rats after treadmill exercise.

## 2 METHODS

### 2.1 Animals

Twelve‐week‐old female Sprague–Dawley rats were obtained from Daehan Biolink (Eumseong, Korea) and housed under controlled conditions with a constant temperature (23 ± 3°C), relative humidity (50 ± 10%), and a 12 h light/dark cycle. The rats were fed a standard rodent diet and provided with purified water ad libitum. The study was conducted following approval from the Institutional Animal Care and Use Committee of the Korea National University of Education, Korea (#KNUE‐202008‐001‐02).

After acclimation to the laboratory environment for 1 week, the rats were randomly divided into three groups: normal control group (CON; *n* = 6), ovariectomized control group (OVX; *n* = 6), and OVX treadmill exercise group (OVX‐EX; *n* = 6). The number of animals used for each group was determined using the Federer formula. Federer's formula is (*n*‐1) (*t*‐1) ≥ 15, where the *n* value is the number of animals per treatment group and *t* value is the number of treatment groups. Based on this formula, three groups were formed, and each group consisted of nine rats. However, according to the Institutional Animal Care and Use Committee's advice, each experiment has to minimize the number of animals. Therefore, the number of rats was reduced to six in each group.

### 2.2 Ovariectomy

Following acclimation, 13‐week‐old rats underwent bilateral ovariectomies using standard procedures. The rats were systemically anesthetized with ether, sterilized (10% povidone‐iodine scrub followed by a 70% alcohol wipe), and underwent hair removal. A 1 cm incision was made in the dorsal center, ligating and removing the ovaries on both sides. Antibiotics (Cafazolin 50 mg/kg) were administered intramuscularly.

### 2.3 Exercise protocol

Rats in the OVX‐EX group were subjected to treadmill exercise 5 days a week for 8 weeks on a zero‐gradient motorized treadmill (Technic Azma Co., Tabriz, Iran). Rats were adapted to their environment for first week prior to aerobic exercise training. During the first week after familiarization, rats exercised by treadmill running at 10 m/min on a 0% gradient for 15 min/day. The exercise intensity was gradually increased to 20 m/min on a 0% gradient for 60 min for a period of 8 weeks. It was gradually improved by about 2 m/min and 6–7 min per week (Ko et al., 2018; Sohroforouzani et al., 2022; Sun et al., 2013).

### 2.4 Analyses of 17β‐estradiol in serum

Blood samples were collected from the jugular vein at 3 and 8 weeks after ovariectomy. The serum was obtained by centrifugation for 20 min at 3000 rpm at 4°C in a microcentrifuge and stored at −20°C until analysis.

Serum estrogen levels were quantified using the 17β‐estradiol ELISA Kit (cat no. ab108667, Abcam) following the manufacturer's instructions. Briefly, 25 μL of blood serum samples, prepared standards, or controls were added in duplicate to a 96‐well plate. Subsequently, 200 μL of the 17 β‐Estradiol‐HRP conjugate was added to each well, followed by incubation at 37°C for 2 h. After incubation, the samples, standards, and controls were aspirated, and wells were washed three times with 300 μL of diluted washing solution. After washing, 100 μL tetramethylbenzidine (TMB) substrate solution was added to each well, and the plate was incubated for 30 min in the dark at room temperature. After incubation period, 100 μL of stop solution was added to all wells in the same order and at the same rate as the substrate solution. Absorbance measurements were performed at 450 nm using a Spectramax M3 plate reader (Molecular Devices) within 30 min of adding the stop solution. The standard curve construction and subsequent analyses were conducted using Microsoft Excel.

### 2.5 Western blot analysis of brain tissues

Twenty‐four hours after the final exercise session, rats were sacrificed under deep anesthesia with diethyl ether. Ether‐soaked cotton ball is held near animal's muzzle in order to maintain anesthesia during perfusion. Rats were humanely euthanized using ether gas overdose. The left hemisphere of the brain was collected after cold saline intracardiac perfusion. Then, the brain tissue was immediately frozen in liquid nitrogen and stored at −80°C until analyses. The brain tissue was homogenized in 10 volumes of radioimmunoprecipitation assay (RIPA) buffer (Thermo Scientific, Waltham, MA, USA) containing protease inhibitors (Sigma‐Aldrich, St. Louis, MO, USA) and phosphatase inhibitors (Sigma‐Aldrich, St. Louis, MO, USA). Western blotting was performed using primary antibodies, followed by incubation with horseradish peroxidase‐conjugated anti‐rabbit secondary antibodies. The antibodies used in this study are listed in Table 1. Band densities were measured and normalized to the actin band density using ImageJ software (NIH, Bethesda, MD, USA).

**TABLE 1:** List of antibodies used in the current study.

	Company	Cat. No.	Dilution
Primary antibodies			
PDGF‐A	Abcam	ab216619	1:1000 (WB)
PDGF‐B	Abcam	ab23914	1:1000 (WB)
PDGF‐AB	Abcam	ab215978	1:300 (IHC)
VEGF	Abcam	ab185238	1:1000 (WB)
Actin	Cell Signal	#5125	1:1000 (WB)
Secondary antibodies			
Goat anti‐rabbit IgG (H + L)	Jackson ImmonoResearch	111–035‐003	1:1000 (WB)
Alexa Fluor™ Plus 488	Invitrogen	A32731	1:300 (IHC)

### 2.6 Quantitative real‐time PCR of brain tissue

Total RNA was isolated from the brain using TRIzol reagent (Invitrogen, Carlsbad, CA, USA) following the manufacturer's instructions. Four micrograms of total RNA were reverse transcribed to generate cDNA using a high‐capacity cDNA Reverse Transcription kit (Applied Biosystems, Foster City, CA, USA), and the resulting cDNA served as a template for subsequent PCR reactions. Quantitative real‐time PCR reactions were conducted using the Power SYBR Green PCR Master Mix (Agilent technology, Santa Clara, CA, USA). Glyceraldehyde 3‐phosphate dehydrogenase (GAPDH) was used as the internal standard to normalize target transcript expression. Primer sets for Ang1, Ang2, and VEGF amplification were employed (Table 2). The reactions were carried out using an AriaMx real‐time PCR system (Agilent Technologies). Triplicate data were analyzed in three independent assays using the comparative Ct method (Yon et al., 2018).

**TABLE 2:** Sequences of the primers used in the current study.

Gene	Forward (5′–3′)	Reverse (5′–3′)	Accession No.
Ang1	GCCACTTGAGAATTACATTGTGG	CGCGGATTTTATGCTCTAATCAACTG	XM_032914910.1
Ang2	GTCTCCCAGCTGACCAGTGGG	TACCACTTGATACCGTTGAAC	XM_032918751.1
GAPDH	GTCGGTGTGAACGGATTTGG	CCACTTTGTCACAAGAGAAGGCA	XM_039103226

### 2.7 Immunohistochemistry in brain sections

For immunohistochemistry, the rat brain was perfusion‐fixed with a 10% paraformaldehyde solution and post‐fixed in the same solution for 48 h. Subsequently, cryoprotection was achieved using 30% sucrose for 72 h. Coronal cryosections, 30‐μm thick and positioned 1.0 mm posterior to bregma, were prepared and processed for immunostaining of PDGF‐AB. Specific antibodies for platelet derived growth factor AB (PDGF‐AB) (1:300, rabbit polyclonal, Abcam) were utilized. Brain sections were incubated with the primary antibodies overnight at 4°C, followed by incubation with secondary antibodies conjugated to Alexa Fluor‐488 (1:300, Molecular Probes) for 2 h at room temperature. This was followed by staining with 4,6‐diamidino‐2‐phenylindole (DAPI) for 10 min. The antibodies used in this study are listed in Table 1. All samples were evaluated immediately after staining and photographed using a fluorescence microscope (EVOS FL Auto2 Cell imaging system; Thermo Fisher Scientific, Waltham, MA, USA).

### 2.8 Statistical analysis

Statistical comparisons between groups were conducted using one‐way analysis of variance (ANOVA), followed by Tukey's multiple comparison test. Pre‐ and post‐exercise data were analyzed using a statistical paired sample *t*‐test. All statistical analyses were performed using the Statistical Package for Social Sciences for Windows (version 22.0; SPSS Inc., Chicago, IL, USA). Statistical significance was set at *p* < 0.05. All data are expressed as mean ± SD.

## 3 RESULTS

### 3.1 17β‐Estradiol levels with serum were increased in OVX rats after treadmill exercise

**FIGURE 1:**
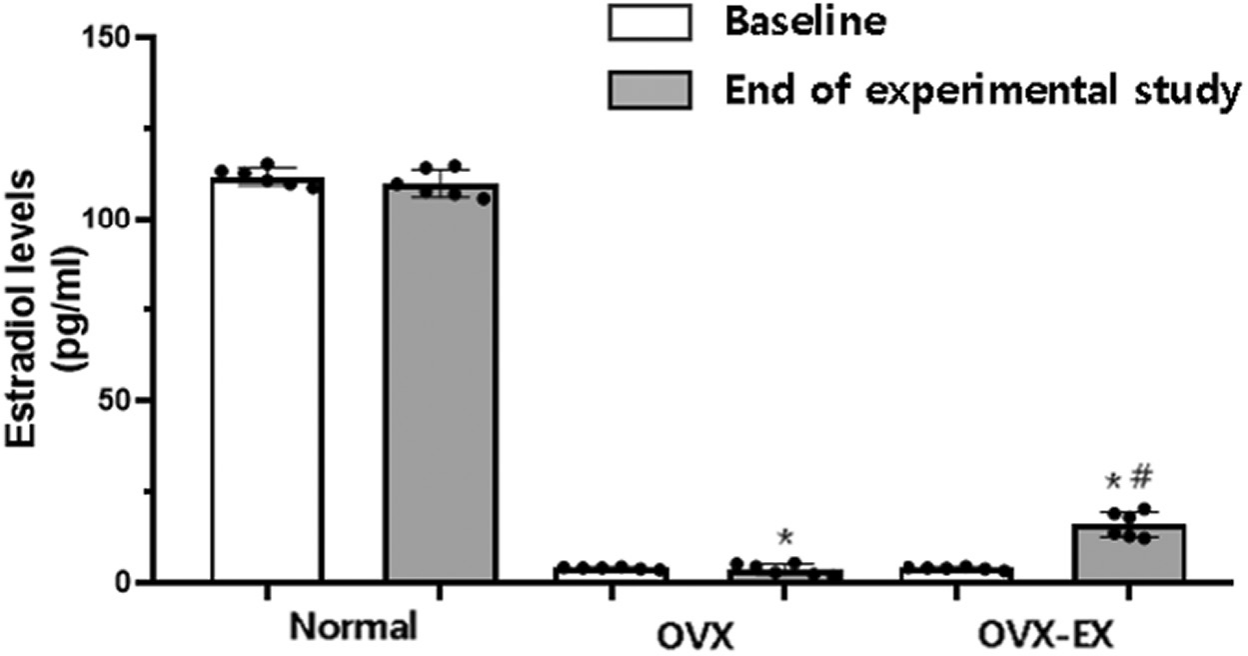
17β‐Estradiol levels in serum at the beginning and end of experiment in different groups (*n* = 6). 17β‐Estradiol levels were analyzed using an ELISA kit. Values are expressed as mean ± SD for six rats. The paired sample *t*‐test was used for the comparison estradiol levels between groups at the beginning and end of experimental study, and the one‐way ANOVA was used for the comparison of the levels between groups at the end of experimental study. Normal, normal control group; OVX, ovariectomized group; OVX‐EX, ovariectomy + exercise group. * Significantly different from the normal control group at the end of study (*p* < 0.05). # Significantly different from baseline and end of experimental study in the OVX‐EX group (*p* < 0.05).

To confirm the efficacy of ovariectomy, 17β‐estradiol levels were measured (Figure 1). Three weeks after surgery recovery, the OVX control group (baseline: 4.21 ± 0.05) and OVX‐EX group (baseline: 4.12 ± 0.27) exhibited significantly reduced 17β‐estradiol levels compared to the normal control group (baseline:112.36 ± 2.54). There was no significant difference in estradiol levels between the normal control group (baseline: 112.36 ± 2.54, end of experimental study: 110.84 ± 3.61, *t* = 0.146, *p* = 0.893) and the OVX control group (baseline: 4.21 ± 0.05, end of experimental study: 3.64 ± 1.59, *t* = 0.629, *p* = 0.564) in terms of pre‐ and post‐exercise levels. However, a significant increase was observed in the OVX‐EX group for pre‐ and post‐exercise levels (baseline: 4.12 ± 0.27, end of experimental study: 16.31 ± 4.02, *t* = 7.720, *p* = 0.000). To sum up, the 17β‐estradiol levels in the OVX‐EX group were lower than those in the normal control group before exercise (*p* < 0.001) but significantly recovered after exercise (baseline: 4.12 ± 0.27, end of experimental study: 16.31 ± 4.02, *p* < 0.001).

### 3.2 Treadmill exercise enhances the expression of the angiogenesis factors, PDGF‐A, PDGF‐B, PDGF‐AB, and VEGF in the brain of ovariectomized rats

**FIGURE 2:**
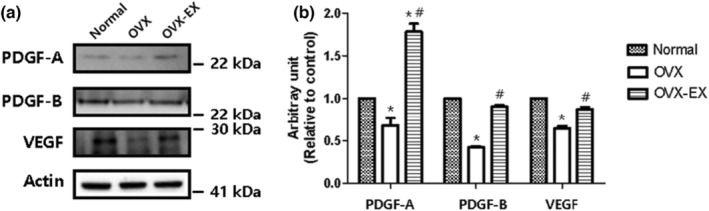
At the end of the experimental study, western blot was performed in the left cortex of rats (*n* = 4). (a) Representative bands of protein related to the angiogenesis factors, platelet derived growth factor (PDGF)‐A and B, and vascular endothermic growth factor (VEGF). (b) The band densities were normalized to that of actin. The one‐way ANOVA was used for the comparison of groups at the end of experimental study. Normal, normal control group; OVX, ovariectomized group; OVX‐EX, ovariectomy + exercise group. * Significantly different from the normal control group (*p* < 0.05). # Significantly different form the OVX control group (*p* < 0.05).

To investigate the effects of estrogen and treadmill exercise on angiogenic factors in the brain, the expression of PDGF‐A, PDGF‐B, and VEGF was confirmed through western blotting and immunohistochemistry (Figure 2a,b). The OVX control group exhibited significantly lower expression of PDGF‐A, PDGF‐B, and VEGF compared to the normal control group (*p* < 0.001). However, at the end of experimental study, the expression of these factors was enhanced in the OVX‐EX group (*p* < 0.001). Interestingly, the expression of PDGF‐A in the OVX‐EX group was higher than that in the normal control group (*p* < 0.001).

**FIGURE 3:**
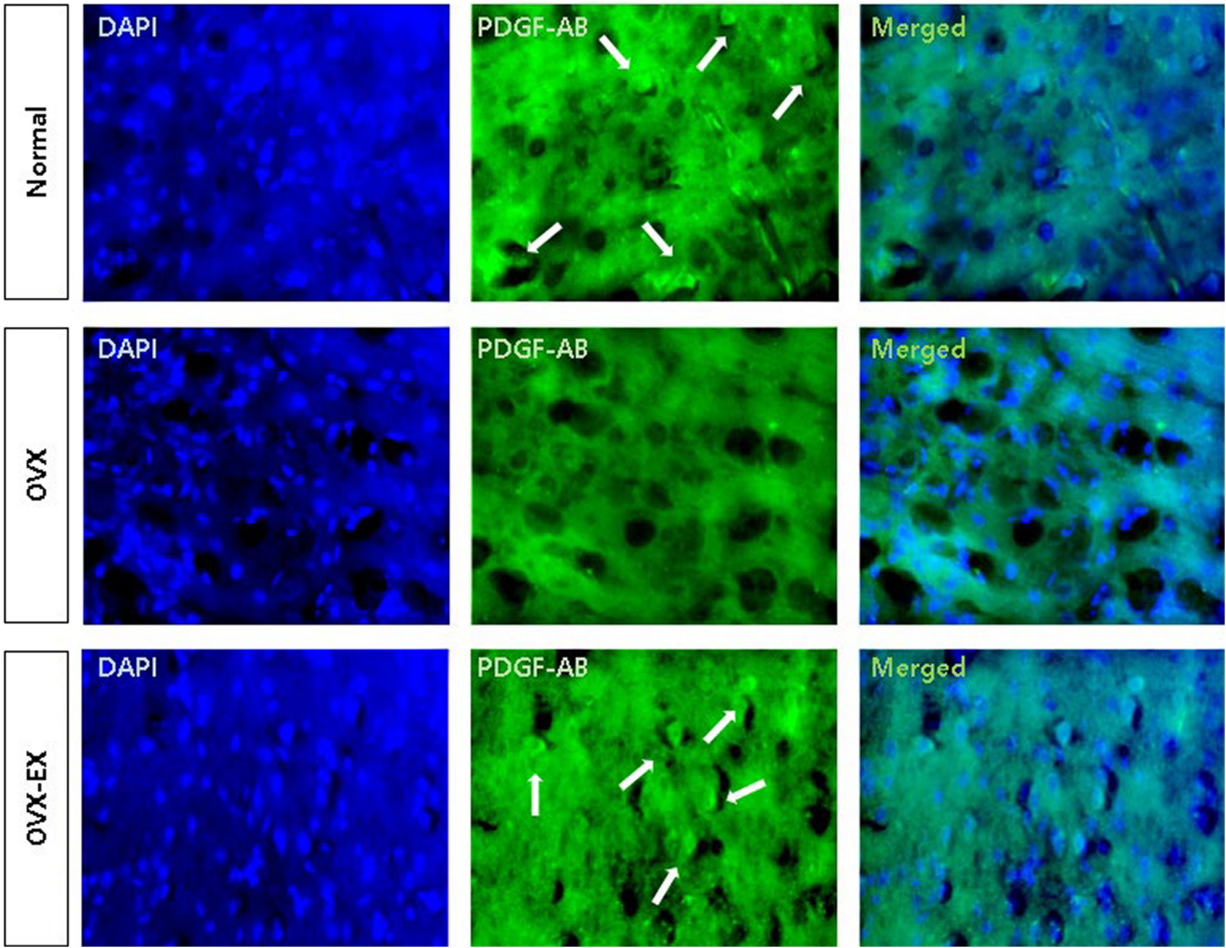
At the end of the experimental study, immunohistochemistry was performed to confirm the expression of platelet derived growth factor (PDGF)‐AB in the cortex of rats (*n* = 2). Representative immunohistochemical images showing the expression of PDGF‐AB in the cortex of rats. Arrowheads represent the area where PDGF‐AB is being expressed. Scale bar = 50 μm, magnification ×200. Normal, normal control group; OVX, ovariectomized group; OVX‐EX, ovariectomy + exercise group.

Figure 3 displays the expression of PDGF‐AB in the rat cortex. Estrogen's impact on the PDGF family was evident, as PDGF‐AB expression was minimal in the cortex of the OVX group compared to the normal control group. However, at the end of experimental study, PDGF‐AB expression increased in the cortex of the OVX‐EX group. These findings indicate that menopause, due to estrogen deficiency, reduces angiogenic factors PDGF‐A and PDGF‐B, but they can be partially restored through exercise.

### 3.3 Treadmill exercise enhances the expression of the angiogenesis factors, Ang1, and Ang2 in the brain of ovariectomized rat

**FIGURE 4:**
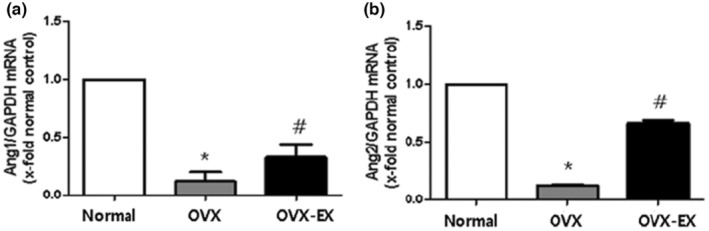
At the end of the experimental study, real‐time PCR was performed in the right cortex of rats (*n* = 4). (a) The expression of Ang1 mRNA in the brain tissues of rats. (b) The expression of Ang2 mRNA in the brain tissues of rats. The one‐way ANOVA was used for the comparison in groups at the end of experimental study. Normal, normal control group; OVX, ovariectomized group; OVX‐EX, ovariectomy + exercise group. * Significantly different from the normal control group (*p* < 0.05). # Significantly different form the OVX control group (*p* < 0.05).

Apart from the PDGF family, Ang1 and Ang2 are important factors in angiogenesis. We performed real‐time PCR on rat brain tissue to examine the effect of exercise on estrogen, Ang1, and Ang2 levels (Figure 4a, b). Ang1 and Ang2 mRNA expression was significantly lower in the OVX control group than in the normal control group (*p* < 0.001). However, 8 weeks of treadmill exercise enhanced the expression of these factors in the OVX‐EX group (*p* < 0.001). These results suggest that menopause, due to estrogen deficiency, decreases angiogenesis factors Ang1 and Ang2, but exercise can lead to partial recovery.

## 4 DISCUSSION

Menopausal women experience various physiological changes due to estrogen deficiency, including dyslipidemia, vascular endothelial cell dysfunction, and increased vascular rigidity, which can contribute to the development and progression of atherosclerotic lesions. Notably, cognitive dysfunction can occur in the blood vessels of the brain. Our previous study has demonstrated the association between estrogen deficiency and cognitive dysfunction, particularly memory loss. (Yoon et al., 2021, 2022). Angiogenesis‐related factors have been proposed as potential candidates for restoring vascular function and alleviating these symptoms.

The PDGF family and VEGF proteins play important roles in angiogenesis by inducing the differentiation, migration, proliferation, and survival of vascular endothelial cells. Additionally, Angs stimulate angiogenesis by promoting the movement and construction of vascular endothelial cells. Menopausal women may have lower expression of these factors due to estrogen deficiency. Our results show that the expression levels of angiogenic factors were lower in the OVX control group than in the normal control group. Estrogen has been reported to modulate angiogenesis, and numerous reports indicate that it increases the expression of PDGF, VEGF, and Ang families. Treatment of TPA‐stimulated cells with E2 caused a 61% and 190% increase in PDGF‐A mRNA at 48 and 96 h, respectively (Shanker et al., 1995). In addition, E2 was found to increase the expression of the VEGFR in human myometrial microvascular endothelial cells (MEC) (Gargett et al., 2002). In addition, E2 was shown to upregulate cerebral Ang1 mRNA by 49% (Ardelt, 2005), and other studies have demonstrated the influence of estrogen on various angiogenesis‐related factors (Tang et al., 2017).

Regular exercise has been shown to have positive effects on the vascular system by improving angiogenesis‐related factors. In the present study, at the end of experimental study, the levels of estradiol and the expression of angiogenesis‐related factors increased in OVX‐EX group compared to that in the OVX control group. We speculate that estradiol increased after exercise in rats with bilateral ovariectomy was because exercise stimulated the adrenal glands to secrete estrogen from the adrenal glands, resulting in estradiol conversion (Hackney & Walz, 2013; Reed et al., 1985). Also, this finding is consistent with those of previous studies showing that exercise upregulates angiogenesis‐related factors. Studies in humans have shown that an acute bout of strenuous exercise leads to a 2–3 fold increase in serum levels of PDGF and VEGF in healthy young sportsmen (Czarkowska‐Paczek et al., 2006). Additionally, exercise was found to elevate PDGF‐B levels in biopsied muscle at 3 h post‐exercise in young men (Trenerry et al., 2011). Similarly, high‐intensity intermittent exercise increases Ang2 expression in muscles at 3 h post‐exercise in young men (Hoier et al., 2013). Angiogenesis is a process involving vessel sprouting, initiated by the transformation of quiescent endothelial cells into tip cells that send out filopodia to guide sprouts in response to the secretion of angiogenesis‐related factors from the hypoxic environment (Gerhardt, 2003). Adjacent cells become stalk cells that proliferate and enhance sprout growth (Hellstrom, 2007). Subsequently, blood flow reactivates, and endothelial cells return to quiescence, releasing basement membrane components and forming tight junctions (Mazzone, 2009). PDGF, VEGF, and Ang are associated with the angiogenesis process. Based on these reports, the increase in the expression of the PDGF family, VEGF, and Ang family induced by exercise may be linked to the influence of hypoxia on angiogenic factors, suggesting that the hypoxic environment during exercise could impact the expression of these factors (Hopkins, 2006). In the event of brain tissue becoming hypoxic, cell function may be compromised, and in some cases, cell death can occur due to energy failure (Larcerte, 2023). To counteract this phenomenon, angiogenesis occurs around damaged tissues, enhancing oxygen transfer to low‐oxygen tissues (Johnson, 2014). As a result, blood flow is augmented to the ischemic area through the newly formed blood vessels, resulting in renewed oxygen supply (Yang, 2020). Based on these results, it is hypothesized that increasing the expression of angiogenesis‐related factors in the brain may counter cognitive dysfunction associated with estrogen deficiency. In fact, in our previous study, we found that cognitive function was improved in the water maze test and passive avoidance test at the same time as angiogenesis‐related factors increased in the ovariectomized exercise group (Yoon et al., 2021).

The current study contributes evidence regarding the effects of aerobic exercise on angiogenesis‐related factors in menopausal women. Further research is needed to elucidate the precise mechanism by which exercise increases the expression of these angiogenesis‐related factors.

## 5 CONCLUSION

The 8‐week treadmill exercise program resulted in a significant increase in the expression of angiogenic factors, including PDGF‐A, PDGF‐B, PDGF‐AB, VEGF, Ang1, and Ang2, in the cortex of OVX models. Both men and women may experience vascular dysfunction in the brain due to estrogen deficiency, but regular aerobic exercise demonstrated the potential to enhance the expression of angiogenic factors, which could be beneficial in overcoming vascular dysfunction in the brain.

## AUTHOR CONTRIBUTIONS

**Eun‐Jung Yoon:** Conceptualization, Data curation, Investigation, Methodology, Validation, Visualization, Writing—original draft. **Dongsun Park:** Conceptualization, Project administration, Supervision, Writing—review and editing. **Eunji Yoon:** Data curation, Investigation, Methodology. **Jiwon Jeong:** Formal analysis, Investigation, Methodology.

## FUNDING INFORMATION

No funding information provided.

## ETHICS STATEMENT

The study was conducted following approval from the Institutional Animal Care and Use Committee of the Korea National University of Education, Korea (#KNUE‐202008‐001‐02).
